# Synchrotron x-ray imaging of pulmonary alveoli in respiration in live intact mice

**DOI:** 10.1038/srep08760

**Published:** 2015-03-04

**Authors:** Soeun Chang, Namseop Kwon, Jinkyung Kim, Yoshiki Kohmura, Tetsuya Ishikawa, Chin Kook Rhee, Jung Ho Je, Akira Tsuda

**Affiliations:** 1 X-ray Imaging Center, Pohang University of Science and Technology, San 31, Hyoja-dong, Pohang, 790-784, Korea; 2 Department of Materials Science and Engineering, Pohang University of Science and Technology, San 31, Hyoja-dong, Pohang, 790-784, Korea; 3 School of Interdisciplinary Bioscience and Bioengineering, Pohang University of Science and Technology, San 31, Hyoja-dong, Pohang, 790-784, Korea; 4 RIKEN SPring-8 Center, 1-1-1 Kouto, Sayo-cho, Sayo, Hyogo, 679-5198, Japan; 5 Division of Pulmonary and Critical Care Medicine, Department of Internal Medicine, Seoul St. Mary's Hospital, Catholic University of Korea, 505 Banpo-dong, Seocho-Gu, Seoul, 137-701, Korea; 6 Harvard School of Public Health, Boston, Massachusetts, USA

## Abstract

Despite nearly a half century of studies, it has not been fully understood how pulmonary alveoli, the elementary gas exchange units in mammalian lungs, inflate and deflate during respiration. Understanding alveolar dynamics is crucial for treating patients with pulmonary diseases. In-vivo, real-time visualization of the alveoli during respiration has been hampered by active lung movement. Previous studies have been therefore limited to alveoli at lung apices or subpleural alveoli under open thorax conditions. Here we report direct and real-time visualization of alveoli of live intact mice during respiration using tracking X-ray microscopy. Our studies, for the first time, determine the alveolar size of normal mice in respiration without positive end expiratory pressure as 58 ± 14 (mean ± s.d.) μm on average, accurately measured in the lung bases as well as the apices. Individual alveoli of normal lungs clearly show heterogeneous inflation from zero to ~25% (6.7 ± 4.7% (mean ± s.d.)) in size. The degree of inflation is higher in the lung bases (8.7 ± 4.3% (mean ± s.d.)) than in the apices (5.7 ± 3.2% (mean ± s.d.)). The fraction of the total tidal volume allocated for alveolar inflation is 34 ± 3.8% (mean ± s.e.m). This study contributes to the better understanding of alveolar dynamics and helps to develop potential treatment options for pulmonary diseases.

Pulmonary alveoli, the elementary gas exchange units of the mammalian lungs, continuously inflate and deflate during respiration. This dynamic behavior of the alveoli significantly influences pulmonary function and stability^1^,^2^,^3^. Understanding alveolar dynamics is therefore crucial not only for studying emphysema or pulmonary edema, but also for treating patients with a variety of pulmonary diseases, such as acute respiratory distress syndrome (ARDS)^4^,^5^, that is a severe form of acute lung injury resulting from sepsis, trauma, or severe pulmonary infections. Patients suffering from these diseases are treated with mechanical ventilation, which eventually has negative side-effects on the lungs, including ventilator induced/associated lung injury (VILI/VALI)^6^,^7^. The visualization of alveolar dynamics has been, however, hampered by active lung movement during respiration.

The average size of the alveoli in live mammalian lungs (which is affected by the changing lung volume associated with breathing) remains undetermined, although this information is fundamental for understanding alveolar dynamics. When mice are sacrificed and the lungs are excised, the size of alveoli undergoes significant changes due to the drastically altered conditions (e.g., intra-thoracic pressure change, removal of surfactant, etc.)^8^,^9^. Moreover, it is not known how much individual alveoli inflate during respiration and whether the inflation is *homogeneous* or *heterogeneous* in live, breathing mammalian lungs. In addition, the fraction of the total tidal volume allocated for the inflation of the alveoli versus the non-alveolar parts of the lungs (e.g., alveolar central ducts) remains undetermined^10^,^11^,^12^,^13^, although these data would be important for determining the optimal total tidal volume when treating ARDS patients with mechanical ventilation.

Real-time imaging of the alveoli is essential for determining the alveolar dynamics during respiration but it has been hindered by active lung movement^14^. Recently, subpleural alveolar clusters in live mice have been studied using intravital microscopy (IVM), optical coherence tomography (OCT), and optical frequency domain imaging (OFDI) under open thorax conditions^15^,^16^,^17^. However, in these studies the alveolar dynamics could be significantly affected by the intrathoracic pressure change once the thorax was opened^16^,^17^,^18^,^8^,^9^. Very recently, alveoli at the upper right lung apices that have a minimum lung movement were studied in live intact mice using tracking X-ray microscopy (TrXM)^19^. However, real-time imaging of alveoli in any other lung regions, in particular, at the lung bases in live intact mice has not been done to date owing to the large respiratory motion.

In this study, we investigate alveolar dynamics not only in the lung apices but also in the bases in live intact mice during respiration, using tracking X-ray microscopy (TrXM II). X-ray imaging based on phase contrast and strongly collimated synchrotron X-rays^20^,^21^ produces images of excellent quality due to strong edge enhancement between different regions^22^,^23^. Furthermore, synchrotron hard X-rays are highly penetrating, enabling us to examine large (> 1 × 1 × 1 mm^3^) regions^24^,^25^,^26^ of the lungs, not limited to subpleural regions. In addition, the projected radiographic images provide accurate information on alveolar size, different from histological images of sliced lung sections. The TrXM II method, which is capable of tracking individual alveoli despite large respiratory motion, allowed us to directly measure the size and degree of inflation of individual alveoli that were located in the lung bases as well as in the apices of live intact mice during respiration.

## Results

Visualization of alveoli described in a previous report^19^ was limited to lung apices and it was not achieved in lung bases, basically due to the active movement of the lungs, which was so big at this lung region that the tracked alveoli were repeatedly in and out of the given field-of-view (FOV) during respiration. For visualization of the alveoli in the bases, therefore, the FOV should be enlarged beyond the movement range of the bases. At the same time, high spatial and temporal resolutions are required to resolve and track the small alveoli in rapid movement during respiration.

**Figure 1:**
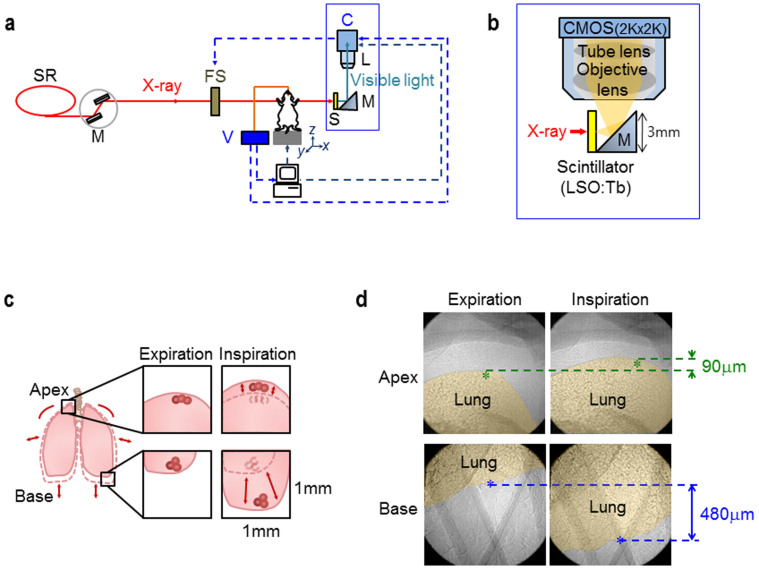
Tracking X-ray microscopy II. (a) Schematic of tracking X-ray microscopy II. The sample stage, a fast shutter, and an X-ray imaging detector system (blue box) were synchronized to a mechanical ventilator. SR: Synchrotron Radiation, M(left): Monochromator, FS: Fast Shutter, V: Ventilator, S: Scintillator, M: Mirror. (b) Schematic of the X-ray imaging detector system. Using a high light yield scintillator of LSO:Tb/LYSO:Ce (4 mm × 4 mm, 3.2 μm thickness), X-ray images were efficiently converted to visible ones. The visible images were then reflected by a small right angle mirror (3 mm height) and magnified by a 20× objective lens coupled with a tube lens for aberration correction before captured by a CMOS camera. (c) Schematic of overall lung movements in the apex top and the base bottom during respiration. (d) Representative microradiographs of the right upper lung apex (yellow) (top panels) and the left lower lung base (yellow) (bottom panels) in a live intact mouse, taken at the ends of expiration and inspiration with a normal tidal volume (160 μl) (Supplementary Video 1).

In the present study, we developed TrXM II (Fig. 1a and b) that allowed us to visualize individual alveoli not only in the lung apices with minimum movement but also in the lung bases with maximum movement (Fig. 1c) during respiration in live intact mice. The key idea was to enlarge FOV to 1 mm × 1 mm, in order to amply cover the area of maximum movements of the bases while providing the high spatial and temporal resolutions of 500 nm (effective pixel size) and 8 ms, respectively, to resolve and track individual alveoli during respiration. Realization of TrXM II was based on a significant improvement in the X-ray imaging detector system (Fig. 1b, blue box in Fig. 1a; see Methods).

Figure 1d shows representative microradiographs of the right upper (RU) lung apex (yellow) (top panels) and the left lower (LL) lung base (yellow) (bottom panels) in a live intact mouse, taken at the ends of expiration and inspiration with a normal tidal volume (160 μl) (Supplementary Video 1). The lung movement is very large (~480 μm) at the base, as indicated by the distance between the blue stars (marking the same location in the two bottom panels), while it is much smaller at the apex (~90 μm, marked by the green stars in the top panels) during respiration. This finding indicated that the FOV (1 mm × 1 mm) was large enough to track individual alveoli in the lung base during respiration.

**Figure 2:**
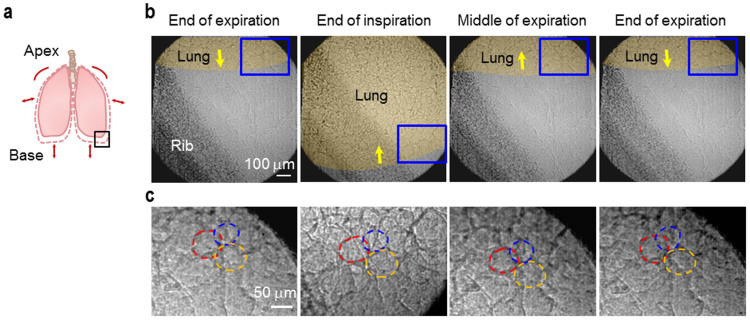
Tracking individual alveoli in the lung base. (a) Schematic of overall lung movement. (b) Representative microradiographs of the lower left lung base (black box of (a)) of a mouse during one normal inspiration (200 ms)-expiration (400 ms) cycle (0, 200, 400, and 600 ms from the left). (c) The magnified regions correspond to blue boxes in (b). Three trackable alveoli (red, orange and blue dashed circles) are demonstrated.

Indeed, TrXM II enabled us to track individual alveoli in the lung base (LL lung base, see black box on Fig. 2a) during respiration, as demonstrated on the 2-dimensional (2-D) microradiographs in Fig. 2c (corresponding to blue boxes on Fig. 2b) by the three alveoli marked by red, orange, and blue dashed circles during one normal inspiration-expiration cycle. In real-time microradiographs, the individual alveoli were sufficiently trackable to measure their sizes by the interference effect from their different movement directions during respiration^19^. This finding indicated that the spatial and temporal resolutions (500 nm and 8 ms, respectively) were high enough to directly measure in *real-time* the size and the degree of inflation of individual alveoli in the lung bases during respiration.

**Figure 3:**
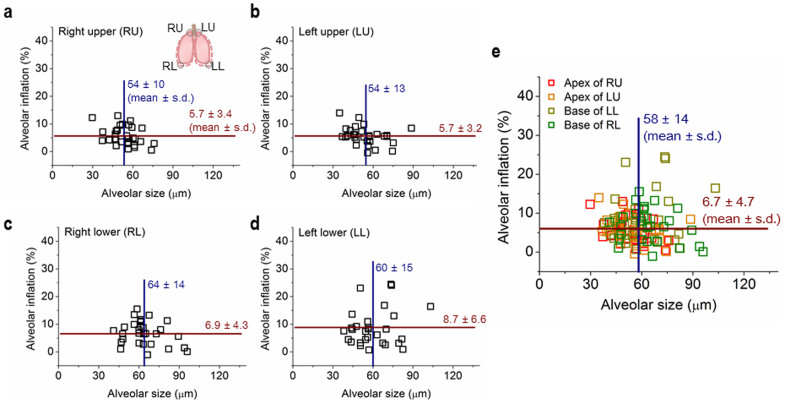
Alveolar size (the major axis of each alveolus) at the end of expiration vs. alveolar inflation at the end of inspiration for the right upper (RU) (a) and the left upper (LU) (b) apices, and the right lower (RL) (c) and the left lower (LL) (d) bases, accurately measured from real-time microradiographs. 24 alveoli per mouse (4 sites, *n* = 5), i.e. a total of 120 alveoli were measured. (e) Overall data for all regions. The navy vertical and brown horizontal lines indicate the average alveolar size and inflation with standard deviations, respectively.

Figure 3 shows alveolar size (at the end of expiration) and alveolar inflation, measured from real-time 2-D microradiographs [see Methods], for the RU (a) and the left upper (LU) (b) apices, and the right lower (RL) (c) and the LL (d) bases. To confirm the validity of our 2-D measurements of alveoli in respiration, we carried out real-time microtomography of the alveoli in respiration in a live intact mouse^19^. The microtomography enabled us to measure the size of individual alveoli of the mouse in 3-D geometry, as demonstrated on Supplementary Fig. 1. Comparison of the alveolar sizes measured in 2-D and 3-D showed a good correlation within 99 ± 2% (Supplementary Fig. 1d), indicating reasonable validity of the 2-D measurements. For statistical analysis, the 2-D measurements were done for 24 alveoli per mouse (4 sites) for 5 mice (Table 1). As seen on the plot of the data for all the regions on Fig. 3e, we found that the average alveolar size of live intact mice was 58 ± 14 (mean ± s.d.) μm (the vertical navy line). The size was on average a little larger in the RL bases (64 ± 14 (mean ± s.d.) μm) than in the RU apices (54 ± 10 (mean ± s.d.) μm) (Table 1 and Fig. 3a–d, *P* = 0.002).

Interestingly, the individual alveoli in normal mice show *heterogeneous* inflation from zero to ~25% in size (Fig. 3a–e; Supplementary Fig. 2). The average inflation, which is 6.7 ± 4.7% (mean ± s.d.) for all (RU, LU, RL, and LL) regions (the horizontal brown line on Fig. 3e), slightly increases from 5.7 ± 3.4 (3.2)% (mean ± s.d.) for the RU (LU) apices to 6.9 ± 4.3% (mean ± s.d., *P* = 0.261) for the RL bases, and to 8.7 ± 6.6% (mean ± s.d., *P* = 0.033) for the LL bases.

**Figure 4:**
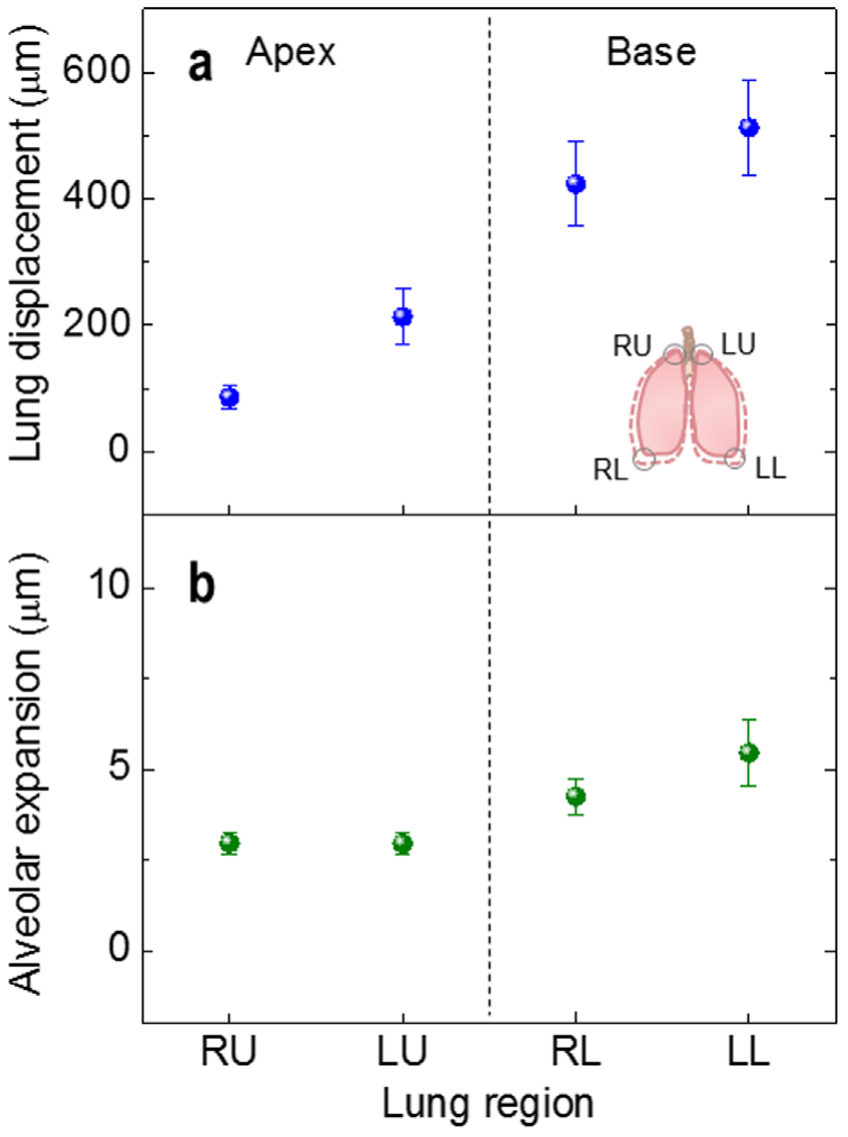
Regional displacements of the pleural surface (lung displacement) (a) and alveolar expansion (b) at the end of inspiration. The lung displacement significantly increases in order of RU, LU, RL, and LL. 24 alveoli per mouse (4 sites, *n* = 5), i.e. a total of 120 alveoli were measured.

Depending on the lung region, the lung movement is significantly different. The smallest is for RU (86 ± 18 μm (mean ± s.e.m.)), increased for LU (214 ± 43 μm (mean ± s.e.m.)) and RL (423 ± 67 μm (mean ± s.e.m.)), and the largest is for LL (513 ± 76 μm (mean ± s.e.m.)), as demonstrated by the regional displacements of the pleural surfaces during respiration in Fig. 4a. The alveolar expansion (Fig. 4b), expressed by absolute expansion of individual alveoli, seems to be influenced by the regional differences in lung movement, increasing from 3.0 ± 0.3 μm (mean ± s.e.m.) in the upper apices to 4.3 ± 0.5 μm (mean ± s.e.m., *P* = 0.034) in the RL bases and further to 5.5 ± 0.9 μm (mean ± s.e.m., *P* = 0.014) in the LL bases.

## Discussion

Despite recent improvements in the experimental techniques used for studying alveolar dynamics, previous works have been limited to studying alveoli at the right upper lung apices that have minimal lung movement, subpleural alveoli under open thorax conditions, or alveoli that have been removed from the body and processed for histology. The strength of TrXM II imaging we use in this study is that it is applicable to dynamic studies of thick living organs in active movements. At the same time it has high spatial and temporal resolution. Here we demonstrate that the high penetrating power of synchrotron hard X-rays enabled us to directly visualize and accurately measure individual alveoli in live intact mice not only in the lung apices but also at the basal lung area during ventilation.

Many reports have been published stating that the lung behaves in a geometrically similar fashion (e.g., Ardila et al., 1974; Weibel 1986^27^,^28^), but all these studies were done in a macroscopic scale. In other words, the data of these publications do not necessarily indicate that the alveolar structure behaves in an isotropic fashion (e.g., Greaves et al., 1986^12^). In fact, we found a large variation, 70% (s.d./mean of Fig. 3e), in alveolar inflation. In other words, even exposed to the same pressure, the inflation of the neighboring alveoli is quite different; this indicates substantial asynchrony at the acinar level. Second, in addition to the significant variations in the degree of alveolar inflation (Fig. 3e), the alveolar duct (which is usually considered to consist of two parts; the central channel and sidewall alveolar pockets) does not appear to expand in an isotropic fashion. Assuming an alveolus can be treated as a 65 ± 3% (mean ± s.e.m) truncated sphere with a sphericity of 0.931 ± 0.022 (mean ± s.e.m.) (Fig. 3b of Chang et al., 2013^19^), alveolar volumes at the ends of expiration and inspiration were estimated (Supplementary Table 1a) based on the measured diameters (Table 1). Furthermore, the average volume changes of alveoli of four lung regions were calculated from the volume difference between inspiration and expiration (Supplementary Table 1b); averaging the values over four lung regions, overall average of alveolar volume change was calculated as ΔV_ave_ = 1.8 ± 0.2 × 10^−5^ μL. (see Supplementary Table 1 for the average volume change of 120 individual alveoli measured over four lung regions in 5 mice). If we assume that the total number of alveoli in mice weighing 22.5 g is about 3 million^29^,^30^,^31^,^32^, the volume change of the sidewall alveolar pockets would be 54 ± 6 μL (mean ± s.e.m.), which is 34 ± 3.8% (mean ± s.e.m.) of the tidal volume (160 μL). The rest (66%) of the tidal volume is accounted for by central channel expansion. The fact that there is a substantial difference in alveolar expansion and central channel expansion indicates that the lung does not expand isotropically at the level of alveolar duct; these data clearly support the observation that the lungs often change their volume but with small change in surface area (e.g., Bachofen et al., 1987; Sera et al., 2013; Fig. 1C of Greaves et al., 1986^12^,^33^,^47^).

A substantial asynchrony at the level of the alveoli must have a significant effect on gas exchange^34^,^35^ and aerosol mixing and deposition^36^,^37^. For instance, since the alveolar airflow is chaotic^38^,^39^, we demonstrated that 10% asynchrony (Miki et al, 1993^40^) can induce a substantial airflow mixing^37^,^41^. Thus, we expect that an even larger variation in alveolar inflation (70%, Fig. 3e) as well as a substantial difference in alveolus/duct expansion could cause significant airflow mixing at the alveolar level enhancing gas exchange and aerosol deposition.

The degree of lung displacement during respiration is presumably associated with the anatomical space available for lung expansion in each anatomical region of the chest of the mouse. As the apical regions are tightly confined by the rib cage, the displacements of the apical regions are small (Fig. 4a). On the other hand, the large movements of the lung at the base are mostly attributed to the active movement of the diaphragm. A slightly larger displacement in the LL than in RL is consistent with this idea, since the motion of the left hemidiaphragm is generally greater than that of the right hemidiaphragm^42^,^43^.

In this study, we successfully applied a novel technology for in-vivo, non-invasive visualization and quantification of alveolar size in live intact mice using TrXM II that enabled tracking individual alveoli in active movements. TrXM-based identification of alveolar dynamics in the RU and LU lung apices and the RL and LL bases revealed that the average alveolar size was 58 ± 14 μm (mean ± s.d.) at functional residual capacity (FRC) and heterogeneous alveolar inflation by 6.7 ± 4.7% (mean ± s.d.) during respiration. The fraction of the total tidal volume allocated for alveolar inflation was estimated as 34 ± 3.8% (mean ± s.e.m). TrXM analysis of the alveoli can open the way to various studies of alveolar dynamics in live intact animals, with future promise to contribute to a better understanding of emphysema and pulmonary edema, and other diseases related to alveolar dysfunction, for instance, VILI in ARDS.

## Methods

The methods were carried out in accordance with the approved guidelines.

### Animal preparation

All experimental protocols were approved by the SPring-8 Experimental Animals Care and Use Committee. Eight-week-old SPF pathogen-free nude mice (BALB/c-nu, body weight: 20–25 g, male, SLC Japan Inc., Japan) were examined. For anesthesia, sodium pentobarbital (50 mg kg^−1^) was injected into peritoneum. Tracheotomy was then performed, followed by the insertion of a catheter (22 G JELCO®I.V. Johnson & Johnson Medical, TX, USA). Ventilation of mice was carried out with room air via the catheter using a ventilator (Inspira-Advanced Safety Ventilator-Pressure Controlled (ASVP), Harvard Apparatus, USA). Physiological respiratory conditions were chosen with an inspiration/expiration ratio of 1:2, a tidal volume of 160 μL/respiration, and a respiratory rate of 100 breaths/min without positive end expiratory pressure. Each mouse was placed in an acrylic tube and fastened in a vertical position for X-ray imaging. After the imaging experiments, all the mice were alive.

### *Real-time* X-ray imaging

Real-time X-ray imaging experiments were performed at the RIKEN Coherent X-ray Optics beamline (BL29XU) at SPring-8 (http://www.spring8.or.jp). The X-ray beam produced by an in-vacuum undulator was monochromatized to 15 keV by a double crystal monochromator, and then transported into the experimental hutch, which was located 98 m from the radiation source. By the high coherency of the monochromatic X-ray, alveolar boundaries, the boundaries between tissues and air, of live intact mice can be clearly visualized by the edge-refraction enhancement^44^,^45^. Taking advantage of the ultra bright synchrotron radiation of SPring-8, the exposure time can be significantly reduced to 8 ms, which allows us to resolve rapidly moving alveoli with little motion blur. Mice were mounted on a high precision motorized stage (Kohzu precision) with the rotational, tilting, and translational resolutions of 0.002°, 0.0009°, and 250 nm, respectively. The transmitted X-ray beam was imaged by the X-ray imaging detector system that is located 15 cm from the mouse (Fig. 1b).

### X-ray imaging detector system (Fig. 1b)

The key goal in developing the X-ray imaging detector system was to enlarge the field of view (FOV) to 1 mm × 1 mm so that it would amply cover the maximum movements of the lung bases of mice while providing the high spatial and temporal resolutions of 500 nm and 8 ms, respectively, to resolve and track individual alveoli during respiration. For this purpose, we applied a CMOS camera (Photron Fastcam SA 2 (Photron, USA; 2048 × 2048 pixels)) in 12-bit (dynamic range) with a maximum frame rate of 86400 f/s. To improve the spatial and temporal resolution, we then adapted the miniaturized conversion (x-rays to visible lights) system^46^ and applied a very high light yield scintillator of LSO:Tb/LYSO:Ce (4 mm × 4 mm, 3.2 μm thickness). After being reflected by a small right angle mirror (3 mm height), the visible images converted by the scintillator were magnified by a 20× objective lens (0.42 N.A.; 20 mm working distance; M Plan Apo 20×; Mitutoyo) coupled with a tube lens for aberration correction before captured by the CMOS camera. The X-ray imaging system enabled us to capture real-time images of mouse lungs in the FOV of 1 mm × 1 mm with the high spatial and temporal resolutions of 500 nm and 125 frames/sec (8 ms), respectively.

### Measurement of alveolar size

Alveolar size was measured both at the end of expiration and at the end of inspiration from 2-D microradiographs. Individual alveoli were first identified with their individual movement paths in 2-D microradiographs during respiration similar to our previous study^19^. Alveolar size was defined here as the maximum diameter of an alveolus, as demonstrated by an orange arrow in Supplementary Fig. 1c. Alveolar inflation is defined as the percentage of the size increase by inspiration.

To confirm the validity of 2-D measurement, the alveolar sizes measured in 2-D and 3-D images were compared. For this, microtomography was performed for a right lower base, using TrXM II with a high-sensitivity camera (PCO.edge, PCO Imaging, Germany), with an exposure time of 20 ms and a resolution of 590 nm/pixel. Each image was taken at the end of expiration during 180° rotation (Supplementary Movie 2). 20 alveolar sizes were measured from 3-D volume-rendered images (Supplementary Fig. 1b) and from 2-D microradiographs (Supplementary Fig. 1c) for identical alveoli, as demonstrated in Supplementary Fig. 1b (an orange arrow) and c (an orange arrow), respectively, for the corresponding alveolus, marked by the arrow head in Supplementary Fig. 1a. A good correlation within 99 ± 2%, calculated by averaging the ratio between the 2D-based size and the 3D-based size, is demonstrated in Supplementary Fig. 1d.

### Statistical analysis

Data were presented as mean ± s.d. or mean ± s.e.m. P-values were determined by performing a two-tailed t-test with data obtained from the right upper apices.

## Author Contributions

S.C., N.K. and J.K. performed experimental work and data analyses. Y.K. and T.I. supported to run synchrotron X-ray imaging experiments in SPring-8. C.K.R. and A.T. performed data analysis and interpretation. J.H.J. supervised the work. J.H.J. and C.K.R. helped to design the experiments. S.C., N.K., J.H.J. and A.T. wrote the manuscript and prepared figures 1–4. All authors reviewed and edited the manuscript.

## Supplementary Material

Supplementary Information Supplementary Information

Supplementary Information Supplementary Video 1

Supplementary Information Supplementary Video 2

## Figures and Tables

**Table 1:** Alveolar size (the major axis of each alveolus) at the ends of expiration and inspiration and alveolar inflation of each region with standard deviation (s.d.). 24 alveoli per mouse (4 sites, *n* = 5), i.e. a total of 120 alveoli were measured

Alveolar size (mean ± s.d μm)				Alveolar inflation (mean ± s.d.%)		
		Right	Left		Right	Left
Apex of Upper lung	Expiration	54 ± 10	54 ± 13	Apex of Upper lung	5.7 ± 3.4	5.7 ± 3.2
	Inspiration	57 ± 10	57 ± 13			
Base of Lower lung	Expiration	64 ± 14	60 ± 15	Base of Lower lung	6.9 ± 4.3	8.7 ± 6.6
	Inspiration	68 ± 14	66 ± 18			

## References

[b1] Cotes J. E., Chinn D. J. & Miller M. R. Lung Function: Physiology, Measurement and Application in Medicine 6th ed. (Blackwell Publishing, 2006).

[b2] Roan E. & Waters C. M. What do we know about mechanical strain in lung alveoli? Am. J. Physiol.-Lung C 301, L625–L635 (2011). 10.1152/ajplung.00105.2011 PMC3213982 21873445

[b3] Hyde D. M., Tyler N. K., Putney L. F., Singh P. & Gundersen H. J. G. Total number and mean size of alveoli in mammalian lung estimated using fractionator sampling and unbiased estimates of the Euler characteristic of alveolar openings. Anat. Rec. Part A 274A, 216–226 (2004). 10.1002/ar.a.20012 14983516

[b4] Schiller H. J. *et al.* Altered alveolar mechanics in the acutely injured lung. Crit. Care. Med. 29, 1049–1055 (2001). 11383531 10.1097/00003246-200105000-00036

[b5] Ware L. B. & Matthay M. A. The acute respiratory distress syndrome. New Engl. J. Med. 342, 1334–1349 (2000). 10793167 10.1056/NEJM200005043421806

[b6] Slutsky A. S. & Ranieri V. M. Ventilator-induced lung injury. New Engl. J. Med. 369, 2126–2136 (2013). 24283226 10.1056/NEJMra1208707

[b7] Gatto L. A., Fluck R. R. Jr & Nieman G. F. Alveolar mechanics in the acutely injured lung: role of alveolar instability in the pathogenesis of ventilator-induced lung injury. Respir. Care 49, 1045–1055 (2004). 15329177

[b8] Weibel E. R. The Pathway for Oxygen Structure and Function in the Mammalian Respiratory System. (Harvard University Press, Cambridge, 1984).

[b9] Bachofen H., Gehr P. & Weibel E. R. Alterations of mechanical properties and morphology in excised rabbit lungs rinsed with a detergent. J. Appl. Physiol. 47, 1002–1010 (1979). 511700 10.1152/jappl.1979.47.5.1002

[b10] Sinclair S. E., Molthen R. C., Haworth S. T., Dawson C. A. & Waters C. M. Airway strain during mechanical ventilation in an intact animal model. Am. J. Resp. Crit. Care 176, 786–794 (2007). 10.1164/rccm.200701-088OC PMC2020825 17626911

[b11] Ford N. L. *et al.* In vivo characterization of lung morphology and function in anesthetized free-breathing mice using micro-computed tomography. J. Appl. Physiol. 102, 2046–2055 (2007). 17255374 10.1152/japplphysiol.00629.2006

[b12] Greaves I. A., Hildebrandt J. & Hoppin F. G. [Micromechanics of the Lung.] Comprehensive Physiology. 217–231 (2011).

[b13] Smaldone G. C. & Mitzner W. Viewpoint: Unresolved mysteries. J. Appl. Physiol. 113, 1945–1947 (2012). 22797308 10.1152/japplphysiol.00545.2012 PMC4587588

[b14] Looney M. R. *et al.* Stabilized imaging of immune surveillance in the mouse lung. Nat. methods 8, 91–96 (2011). 21151136 10.1038/nmeth.1543 PMC3076005

[b15] Mertens M. *et al.* Alveolar dynamics in acute lung injury: heterogeneous distension rather than cyclic opening and collapse. Crit. Care Med. 37, 2604–2611 (2009). 19623041 10.1097/CCM.0b013e3181a5544d

[b16] Meissner S., Tabuchi A., Mertens M., Kuebler W. M. & Koch E. Virtual four-dimensional imaging of lung parenchyma by optical coherence tomography in mice. J. Biomed. Opt. 15, 036016 (2010). 20615018 10.1117/1.3425654

[b17] Namati E. *et al.* Four-dimensional visualization of subpleural alveolar dynamics in vivo during uninterrupted mechanical ventilation of living swine. Biomed. Opt. Express 4, 2492–2506 (2013). 24298409 10.1364/BOE.4.002492 PMC3829543

[b18] Carney D., DiRocco J. & Nieman G. Dynamic alveolar mechanics and ventilator-induced lung injury. Crit. Care Med. 33, S122–S128 (2005). 15753717 10.1097/01.ccm.0000155928.95341.bc

[b19] Chang S. *et al.* Tracking X-ray microscopy for alveolar dynamics in live intact mice. Sci. Rep. 3, 1304 (2013). 23416838 10.1038/srep01304 PMC3575013

[b20] Hwu Y. *et al.* Imaging cells and tissues with refractive index radiology. Biophys. J. 87, 4180–4187 (2004). 15465870 10.1529/biophysj.103.034991 PMC1304927

[b21] Hwu Y. *et al.* Synchrotron microangiography with no contrast agent. Phys. Med. Biol. 49, 501–508 (2004). 15005160 10.1088/0031-9155/49/4/002

[b22] Hwu Y., Tsai W. L., Groso A., Margaritondo G. & Je J. H. Coherence-enhanced synchrotron radiology: simple theory and practical applications. J. Phys. D Appl. Phys. 35, R105–R120 (2002).

[b23] Kim J. *et al.* Altered branching patterns of Purkinje cells in mouse model for cortical development disorder. Sci. Rep. 1, 122 (2011). 22355639 10.1038/srep00122 PMC3216603

[b24] Kim J. *et al.* Growth patterns for acervuli in human pineal gland. Sci. Rep. 2, 984 (2012). 23248747 10.1038/srep00984 PMC3523289

[b25] Kim J., Choi Y. H., Chang S., Kim K. T. & Je J. H. Defective folliculogenesis in female mice lacking Vaccinia-related kinase 1. Sci. Rep. 2, 468 (2012). 22741057 10.1038/srep00468 PMC3384087

[b26] Margaritondo G., Hwu Y. & Je J. H. Synchrotron light in medical and materials science radiology. Riv. Nuovo Cimento 27, 1–40 (2004).

[b27] Ardila R., Horie T. & Hildebrandt J. Macroscopic isotropy of lung expansion. Resp. Physiol. 20, 105–115 (1974). 10.1016/0034-5687(74)90100-5 4826745

[b28] Weibel E. R. [Functional Morphology of Lung Parenchyma.] Comprehensive Physiology [Fishman, A. P. (ed.)] (Bethesda, MD, Am. Physiol. Soc. 1986).

[b29] Knust J., Ochs M., Gundersen H. J. & Nyengaard J. R. Stereological estimates of alveolar number and size and capillary length and surface area in mice lungs. Anat. Rec. 292, 113–122 (2009). 10.1002/ar.20747 19115381

[b30] Vasilescu D. M. *et al.* Assessment of morphometry of pulmonary acini in mouse lungs by nondestructive imaging using multiscale microcomputed tomography. Proc. Natl. Acad. Sci. U.S.A. 109, 17105–17110 (2012). 23027935 10.1073/pnas.1215112109 PMC3479519

[b31] Vasilescu D. M. *et al.* Stereological assessment of mouse lung parenchyma via nondestructive, multiscale micro-CT imaging validated by light microscopic histology. J. Appl. Physiol. 114, 716–724. (2012) 23264542 10.1152/japplphysiol.00855.2012 PMC3615598

[b32] Fehrenbach H. *et al.* Neoalveolarisation contributes to compensatory lung growth following pneumonectomy in mice. Eur. Respir. J. 31, 515–522 (2008). 18032439 10.1183/09031936.00109407

[b33] Bachofen H., Schurch S., Urbinelli M. & Weibel E. R. Relations among alveolar surface tension, surface area, volume, and recoil pressure. J. Appl. Physiol. 62, 1878–1887 (1987). 3597262 10.1152/jappl.1987.62.5.1878

[b34] Wilson T. A., Olson L. E. & Rodarte J. R. Effect of variable parenchymal expansion on gas mixing. J. Appl. Physiol. 62, 634–639 (1987). 3558222 10.1152/jappl.1987.62.2.634

[b35] Swan A. J. & Tawhai M. H. Evidence for minimal oxygen heterogeneity in the healthy human pulmonary acinus. J. Appl. Physiol. 110, 528–537 (2011). 21071589 10.1152/japplphysiol.00888.2010 PMC3043789

[b36] Haber S., Butler J. P., Brenner H., Emanuel I. & Tsuda A. Shear flow over a self-similar expanding pulmonary alveolus during rhythmical breathing. J. Fluid Mech. 405, 243–268 (2000).

[b37] Tsuda A., Henry F. S. & Butler J. P. Particle transport and deposition: basic physics of particle kinetics. Compr. Physiol. 3, 1437–1471 (2013). 24265235 10.1002/cphy.c100085 PMC4398662

[b38] Tsuda A., Henry F. S. & Butler J. P. Chaotic mixing of alveolated duct flow in rhythmically expanding pulmonary acinus. J. Appl. Physiol. 79, 1055–1063 (1995). 8567502 10.1152/jappl.1995.79.3.1055

[b39] Tsuda A., Laine-Pearson F. E. & Hydon P. E. Why Chaotic mixing of particles is inevitable in the deep lung. J. Theor. Biol. 286, 57–66 (2011) 21801733 10.1016/j.jtbi.2011.06.038 PMC3386790

[b40] Miki H., Butler J. P., Rogers R. A. & Lehr J. L. Geometric hysteresis in pulmonary surface-to-volume ratio during tidal breathing. J. Appl. Physiol. 75, 1630–1636 (1993). 8282613 10.1152/jappl.1993.75.4.1630

[b41] Tsuda A., Otani Y. & Butler J. P. Acinar flow irreversibility caused by perturbations in reversible alveolar wall motion. J. Appl. Physiol. 86, 977–984 (1999). 10066713 10.1152/jappl.1999.86.3.977

[b42] Gerscovich E. O. *et al.* Ultrasonographic Evaluation of Diaphragmatic Motion. J. Ultras. Med. 20, 597–604 (2001). 10.7863/jum.2001.20.6.597 11400933

[b43] Kharma N. Dysfunction of the diaphragm: imaging as a diagnostic tool. Curr. Opin. Pulm. Med. 19, 394–398 (2013). 23715292 10.1097/MCP.0b013e3283621b49

[b44] Monfraix S. *et al.* Quantitative measurement of regional lung gas volume by synchrotron radiation computed tomography. Phys. Med. Biol. 50, 1–11 (2005). 15715418 10.1088/0031-9155/50/1/001

[b45] Dubsky S., Hooper S. B., Siu K. K. W. & Fouras A. Synchrotron-based dynamic computed tomography of tissue motion for regional lung function measurement. J. R. Soc. Interface 9, 2213–2224 (2012). 22491972 10.1098/rsif.2012.0116 PMC3405755

[b46] Jung J. W. *et al.* Fast microtomography using bright monochromatic x-rays. Rev. Sci. Instrum. 83, 0937041–4 (2012). 10.1063/1.4751853 23020380

[b47] Sera T. *et al.* Murine pulmonary acinar mechanics during quasi-static inflation using synchrotron refraction-enhanced computed tomography. J. Appl. Physiol. 115, 219–228 (2013). 23661619 10.1152/japplphysiol.01105.2012

