# Supplementary material for: Synchrotron x-ray imaging of pulmonary alveoli in respiration in live intact mice
Source: Sci Rep. 2015 Mar 4;5:8760. doi: 10.1038/srep08760 (PMC4348649; doi:10.1038/srep08760)
Supplement: Supplementary Information [file srep08760-s1.doc]

SUPPLEMENTARY INFORMATION

**Synchrotron x-ray imaging of pulmonary alveoli in respiration in live intact mice**

Soeun Chang, Namseop Kwon, Jinkyung Kim, Yoshiki Kohmura, Tetsuya Ishikawa, Chin Kook Rhee, Jung Ho Je, and Akira Tsuda

| Supplementary Figure 1 | Comparison of alveolar sizes measured in 2-D and 3-D |
| --- | --- |
| Supplementary Figure 2 | Histograms of alveolar inflation |
| Supplementary Table 1 | Average alveolar volumes at the ends of expiration and inspiration and average volume change |

*Note: Supplementary Videos 1 and 2 are available on the Scientific Reports website.*

**Video Legends**

**Supplementary Video 1.**

Microradiographs of a left lower lung base in a live intact mouse, taken during ventilation with a normal tidal volume (160 μl).

**Supplementary Video 2.**

Microradiographs of a mouse, taken at the end of expiration during 180° rotation with a normal tidal volume (160 μl).

**Supplementary Figure 1**


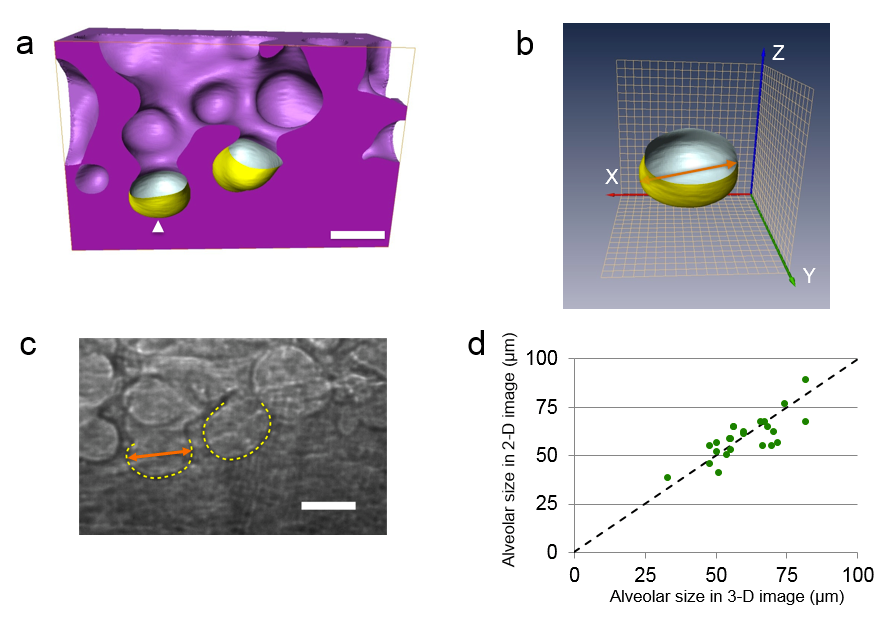


**Comparison of alveolar sizes measured in 2-D and 3-D.** (a) An 3-D volume rendered image of a lung base region of a mouse. (b) The alveolus marked by the white arrow head in (a) is drawn in 3-D coordinates. (c) One 2-D microradiograph corresponding to (a). Yellow dashed lines indicate the two alveoli in (a). (d) Alveolar sizes are plotted in 2-D and 3-D. (Scale bar: 50 μm)

**Supplementary Figure 2**


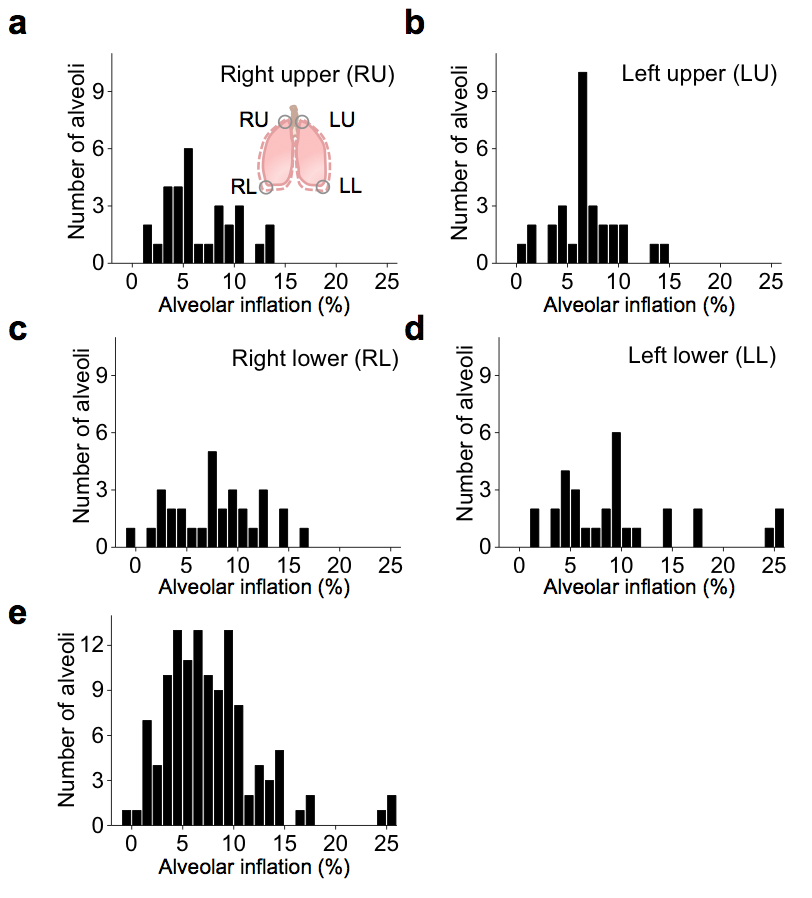


**Histograms of alveolar inflation** for the right upper (a), the left upper (b), the right lower (c), the left lower (d), and the overall (e) regions of mice lungs.

**Supplementary Table 1**

**a**

**b**

| Alveolar volume (mean ± s.e.m. × 10^-5^ μL) | | | |
| --- | --- | --- | --- |
|  | | Right | Left |
| Apex of Upper lung | Expiration | 5.9 ± 0.6 | 6.4 ± 0.9 |
|  | Inspiration | 6.8 ± 0.6 | 7.4 ± 1.1 |
| Base of Lower lung | Expiration | 10.3 ± 1.3 | 8.9 ± 1.3 |
|  | Inspiration | 12.3 ± 1.4 | 12.0 ± 2.1 |

| Average volume change (ΔV)  (mean ± s.e.m. × 10^-5^ μL) | | |
| --- | --- | --- |
|  | Right | Left |
| Apex of Upper lung | 1.0 ± 0.1 | 1.1 ± 0.2 |
| Base of Lower lung | 2.0 ± 0.3 | 3.1 ± 0.9 |
| Over the four regions | 1.8 ± 0.2 | |

Average alveolar volumes at the ends of expiration and inspiration (a) and average volume change (b) in each region.
